# Five-Year Trends in US Children’s Health and Well-being, 2016-2020

**DOI:** 10.1001/jamapediatrics.2022.0056

**Published:** 2022-03-14

**Authors:** Lydie A. Lebrun-Harris, Reem M. Ghandour, Michael D. Kogan, Michael D. Warren

**Affiliations:** 1 US Department of Health and Human Services, Health Resources and Services Administration, Maternal and Child Health Bureau

## Abstract

**Importance:**

Ensuring the well-being of the 73 million children in the United States is critical for improving the nation’s health and influencing children’s long-term outcomes as they grow into adults.

**Objective:**

To examine recent trends in children’s health-related measures, including significant changes between 2019 and 2020 that might be attributed to the COVID-19 pandemic.

**Design, Setting, and Participants:**

Annual data were examined from the National Survey of Children’s Health (2016-2020), a population-based, nationally representative survey of randomly selected children. Participants were children from birth to age 17 years living in noninstitution settings in all 50 states and the District of Columbia whose parent or caregiver responded to an address-based survey by mail or web. Weighted prevalence estimates account for probability of selection and nonresponse. Adjusted logistic regression models tested for significant trends over time.

**Main Outcomes and Measures:**

Diverse measures pertaining to children’s current health conditions, positive health behaviors, health care access and utilization, and family well-being and stressors.

**Results:**

A total of 174 551 children were included (annual range = 21 599 to 50 212). Between 2016 and 2020, there were increases in anxiety (7.1% [95% CI, 6.6-7.6] to 9.2% [95% CI, 8.6-9.8]; +29%; trend *P* < .001) and depression (3.1% [95% CI, 2.9-3.5] to 4.0% [95% CI, 3.6-4.5]; +27%; trend *P* < .001). There were also decreases in daily physical activity (24.2% [95% CI, 23.1-25.3] to 19.8% [95% CI, 18.9-20.8]; −18%; trend *P* < .001), parent or caregiver mental health (69.8% [95% CI, 68.9-70.8] to 66.3% [95% CI, 65.3-67.3]; −5%; trend *P* < .001), and coping with parenting demands (67.2% [95% CI, 66.3-68.1] to 59.9% [95% CI, 58.8-60.9]; −11%; trend *P* < .001). In addition, from 2019 to 2020, there were increases in behavior or conduct problems (6.7% [95% CI, 6.1-7.4] to 8.1% [95% CI, 7.5-8.8]; +21%; *P* = .001) and child care disruptions affecting parental employment (9.4% [95% CI, 8.0-10.9] to 12.6% [95% CI, 11.2-14.1]; +34%; trend *P* = .001) as well as decreases in preventive medical visits (81.0% [95% CI, 79.7-82.3] to 74.1% [95% CI, 72.9-75.3]; −9%; trend *P* < .001).

**Conclusions and Relevance:**

Recent trends point to several areas of concern that can inform future research, clinical care, policy decision making, and programmatic investments to improve the health and well-being of children and their families. More analyses are needed to elucidate varying patterns within subpopulations of interest.

## Key Points

**Question:**

What are the recent trends in children’s health, including significant changes that might be attributed to the COVID-19 pandemic?

**Findings:**

Between 2016 and 2020, there were significant increases in children’s diagnosed anxiety and depression, decreases in physical activity, and decreases in caregiver mental and emotional well-being and coping with parenting demands. After the onset of the pandemic specifically, there were significant year-over-year increases in children’s diagnosed behavioral or conduct problems, decreases in preventive medical care visits, increases in unmet health care needs, and increases in the proportion of young children whose parents quit, declined, or changed jobs because of child care problems.

**Meaning:**

Study findings point to several areas of concern that can inform future research, clinical care, policy decision making, and programmatic investments to improve the health and well-being of children and their families.

## Teaser

This survey study examines data from the US National Survey of Children’s Health to identify trends in children’s health from 2016 to 2020, including significant changes that might be attributed to the COVID-19 pandemic.

## Introduction

In 2019, there were 73 million children aged 0 to 17 years living in the United States, which is 22% of the population.^1^ Improving the country’s overall health status requires a focus on the well-being of children and their families, as this critical period can have lifelong health effects.^2,3,4^ Although children are generally perceived to be healthy, significant proportions are affected by various health conditions, including an estimated 1 in 5 children who have special health care needs.^5^ Recent studies have documented increasing rates of developmental disabilities, diabetes, and overweight and obesity.^6,7,8^ Healthy People 2030, the federal initiative that tracks data-driven objectives to improve the nation’s health, highlights several avenues to improve children’s health and well-being: ensuring access to timely health care services, promoting positive health behaviors, and strengthening supportive family relationships.^9^ An assessment of related measures and recent trends in children’s health and health-related factors is needed to inform interventions and policy priorities. The COVID-19 pandemic has further underscored the need to monitor children’s health. In addition to the direct effects of the pandemic on pediatric populations (infection, hospitalization, and deaths),^10^ the indirect effects have been pervasive, ranging from family economic hardships to reduced physical activity and increased screen time.^11,12,13,14,15^

The recent release of the 2020 National Survey of Children’s Health (NSCH) offers an opportunity to examine 5-year trends in children’s well-being, including an exploration of potential effects of the COVID-19 pandemic. The purpose of this study was to assess changes over time in several domains: children’s health conditions, positive health behaviors, access to and utilization of health care services, and family well-being and stressors. We sought to answer (1) What are the recent trends across various children’s health-related measures? (2) Were there significant changes between 2019 and 2020, which might be attributed to the COVID-19 pandemic?

## Methods

### Data Source

Data came from the NSCH, a nationally representative survey of children from birth to age 17 years living in noninstitution settings in the 50 states and the District of Columbia. Data are collected annually between June or July and January from parents or other primary caregivers through web- or paper-based questionnaires. The NSCH is funded and directed by Maternal and Child Health Bureau of the Health Resources and Services Administration and fielded by the US Census Bureau. More information about the survey methodology is available elsewhere.^16,17,18^ The study used existing publicly available and deidentified data; therefore, it did not qualify as human subjects research and did not require institutional review board review.^19^

We analyzed data from the years 2016 through 2020. The 2020 NSCH was fielded from June 2020 to January 2021; data collection was not disrupted by the COVID-19 pandemic.^18^ Overall response rates ranged from 37% to 43% depending on year. Response rates are adversely affected by the 40% to 50% of sampled addresses that cannot be confirmed as occupied households yet are included in the denominator for response rate calculations. Interview completion rates, which represent the proportion of confirmed, occupied households with children who completed the survey, ranged from 70% to 81%. The analytic sample included children aged birth to 17 years, with measures of interest further restricted to narrower age groups as developmentally or clinically appropriate. The combined NSCH sample size for 2016 to 2020 included 174 551 children (annual range: 21 599-50 212).

### Measures

Guided by Healthy People 2030, we considered a diverse set of measures related to children’s health. Specifically, we examined common health conditions, positive health behaviors, health care access and utilization, and family well-being and stressors (eTable 1 in the Supplement). For children’s health conditions, we examined 9 current health problems (asthma, headaches/migraines, anxiety problems, depression, behavioral/conduct problems, autism, attention-deficit/hyperactivity disorder [ADHD], decayed teeth/cavities, overweight/obesity) as well as presence of special health care needs.^20^

For positive health behaviors, we considered adequate sleep, daily reading to young children, and daily physical activity for school-aged children. For health care access, we examined current uninsurance, insurance adequacy and continuity, problems paying child’s medical bills, unmet health care needs, frustration obtaining health services for child, and having a usual source of sick care. For health care utilization, we included past-year receipt of preventive medical visits, preventive dental visits, specialty care, mental health treatment or counseling, and developmental screening. For preventive medical visits, we excluded data from 2018 because of a wording change in the survey item for that year.

Regarding family well-being and stressors, we considered primary caregiver physical and mental health status; perceptions of coping with the demands of raising children; quitting, declining, or changing jobs because of child care problems; and household food insufficiency. We also examined selected adverse childhood experiences during the child’s lifetime.

### Statistical Analysis

We pooled 5 years of data into a single datafile, which included a variable for survey year. We produced weighted unadjusted prevalence estimates, along with 95% CIs, for each year between 2016 and 2020 (eTable 2 in the Supplement contains annual estimates from 2016-2020 inclusive as well as estimated population frequencies). We calculated absolute and relative differences to determine the magnitude of changes over time. Relative difference, presented as a percentage, is the absolute difference divided by the prevalence in the referent category (2016) multiplied by 100.

For trend analyses, we ran logistic regression models with survey year treated as a continuous variable and tested for linear trends to assess whether changes over time were statistically significant after controlling for demographic variables. We adjusted trend models for child age (0-5, 6-11, and 12-17 years), sex (male, female), race and ethnicity, and household income (<200% and ≥200% federal poverty level) to control for the possibility that changing demographics among the US child population might be driving observed changes.

Race and ethnicity were self-reported during the survey and subsequently categorized as Hispanic, non-Hispanic Black, non-Hispanic White, or non-Hispanic other or multiple race. We assessed race and ethnicity because of their established association with various indicators of children’s health and health care.

To examine changes in trends that might have occurred with the onset of the COVID-19 pandemic, we produced 3 sets of models: 1 to test trends over the entire 5-year period (2016-2020), 1 to test trends in the 4 years before the pandemic (2016-2019), and 1 to compare prevalence estimates between 2019 and 2020. For estimates that showed no significant changes prepandemic, we conducted sensitivity analyses by pooling data across the 4 years of 2016 to 2019 to increase power and assessed whether results changed when comparing the pooled-data period vs 2020.

Analyses accounted for complex survey sampling design and were weighted to produce estimates that were nationally representative of US children living in noninstitutional settings, using Stata MP version 15 (StataCorp).^21^ Statistical significance was assessed using a 2-sided *P* value threshold of .05. As this was a descriptive, exploratory analysis, no adjustments were made for multiple comparisons. Observations with missing or unknown data were dropped from the analysis. Sex (0.1% missing), race (0.4% missing), and ethnicity (0.5% missing) were imputed using hot-deck imputation, and household income (17.8% missing) was multiply imputed using regression methods. More information is available elsewhere on imputation methods.^22^

## Results

### Children’s Health Conditions and Positive Health Behaviors

**Figure 1.**
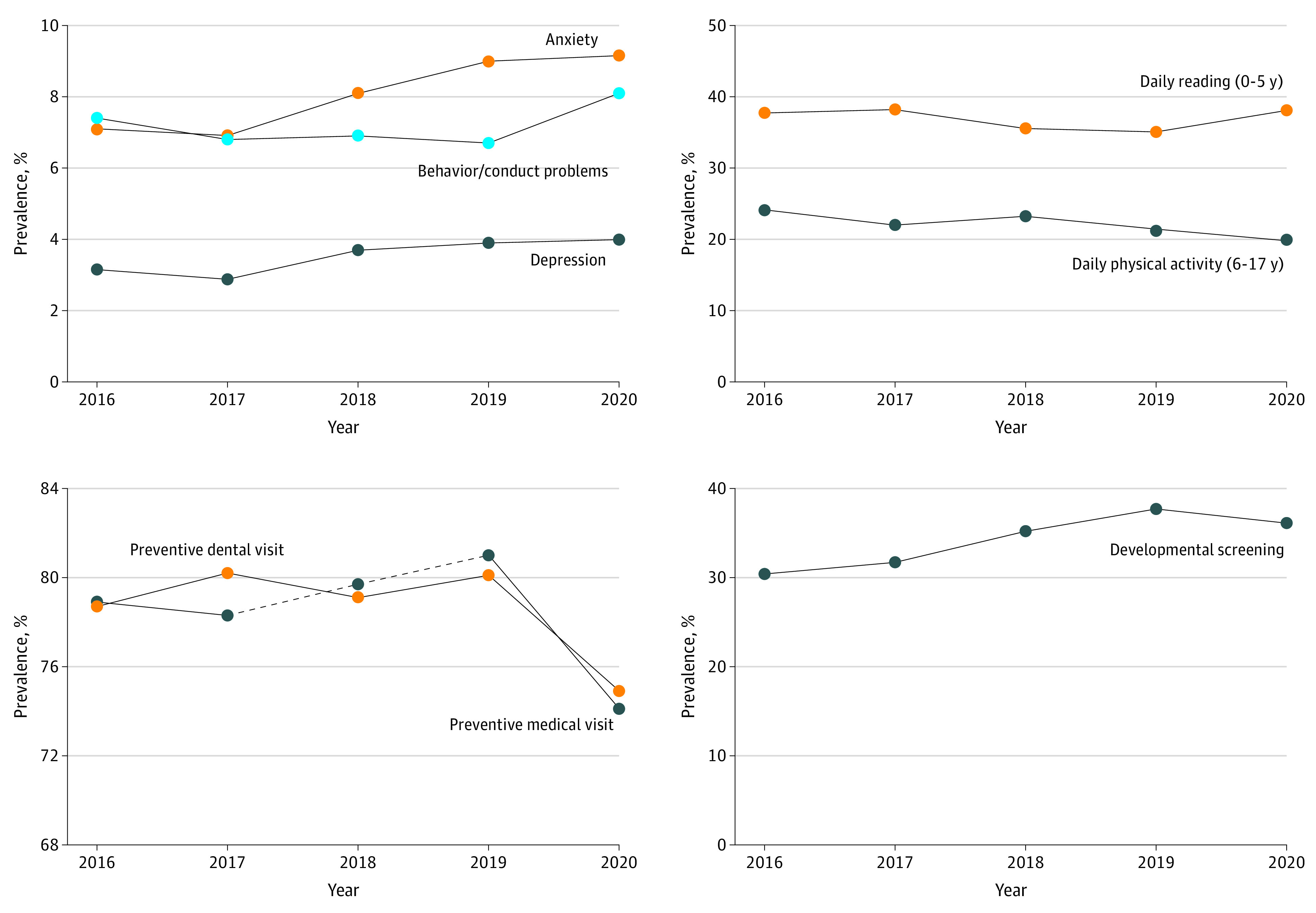
Trends in Selected Measures of Children’s Health Conditions, Positive Health Behaviors, and Health Care Utilization, 2016-2020

Between 2016 and 2020, there was a significant decrease in asthma (8.4% [95% CI, 7.9-9.0] to 7.2% [95% CI, 6.7-7.7]; 14% decrease; *P* = .03) as well as significant increases in anxiety problems (7.1% [95% CI, 6.6-7.6] to 9.2% [95% CI, 8.6-9.8]; 29% increase; *P* < .001) and depression (3.1% [95% CI, 2.9-3.5] to 4.0% [95% CI, 3.6-4.5], 27% increase; *P* < .001]) (Table 1 and Figure 1). Increases in anxiety and depression were evident before the onset of the pandemic (2016-2019), with modest but statistically nonsignificant continuations of these trends in 2020. There was a significant increase in behavior or conduct problems between 2019 and 2020 (6.7% [95% CI, 6.1-7.4] to 8.1% [95% CI, 7.5-8.8]; 21% increase; *P* = .001). There was also a significant 5-year decrease in the proportion of school-aged children getting at least 60 minutes of daily physical activity (24.2% [95% CI, 23.1-25.3] to 19.8% [95% CI, 18.9-20.8]; 18% decrease; *P* < .001), a trend that began before the pandemic and continued in 2020. Prior to the pandemic, there was a significant decrease in the proportion of young children who were read to daily (37.7% [95% CI, 36.1-39.4] to 35.1% [95% CI, 33.1-37.1]; 7% decrease; *P* = .01); however, the prevalence increased again in 2020, resulting in no overall change. There were no statistically significant changes over time for the prevalence of headaches or migraines, autism, ADHD, overweight and obesity, decayed teeth and cavities, special health care needs, or adequate sleep.

**Table 1.** Unadjusted Prevalence and Adjusted Trends for Children’s Current Health Conditions and Positive Health Behaviors, 2016-2020 (N = 174 551)

	Weighted prevalence, % (95% CI)^a^			5-y Trends (2016-2020)			Before COVID-19 (2016-2019)			COVID-19 era (2020 vs 2019)		
	2016 (n = 50 212)	2019 (n = 29 433)	2020 (n = 42 777)									
				Absolute difference	Relative difference	*P* value for trend (adj)^b^	Absolute difference	Relative difference	*P* value for trend (adj)^b^	Absolute difference	Relative difference	*P* value for trend (adj)^b^
**Current health conditions**												
Asthma	8.4 (7.9-9.0)	7.8 (7.2-8.5)	7.2 (6.7-7.7)	−1.2	−14.2	.03	−0.6	−7.3	.27	−0.6	−7.5	.15
Headaches/migraines (3-17 y)	3.5 (3.1-4.0)	3.3 (2.8-3.8)	3.0 (2.6-3.4)	−0.6	−15.7	.21	−0.3	−7.5	.70	−0.3	−8.9	.38
Anxiety problems (3-17 y)	7.1 (6.6-7.6)	9.0 (8.3-9.7)	9.2 (8.6-9.8)	2.1	28.9	<.001	1.9	26.7	<.001	0.2	1.7	.69
Depression (3-17 y)	3.1 (2.9-3.5)	3.9 (3.4-4.4)	4.0 (3.6-4.5)	0.8	26.7	<.001	0.7	23.8	<.001	0.1	2.4	.77
Behavioral/conduct problems (3-17 y)	7.4 (6.9-7.9)	6.7 (6.1-7.4)	8.1 (7.5-8.8)	0.7	10.2	.06	−0.6	−8.6	.30	1.4	20.6	.001
Autism (3-17 y)	2.5 (2.2-2.8)	3.1 (2.7-3.6)	2.7 (2.4-3.1)	0.2	9.4	.38	0.6	24.6	.10	−0.4	−12.1	.19
ADHD (3-17 y)	8.9 (8.4-9.4)	8.6 (8.0-9.3)	9.3 (8.7-9.9)	0.4	4.0	.27	−0.3	−2.9	.84	0.6	7.1	.14
Decayed teeth/cavities (1-17 y)	11.7 (11.0-12.4)	11.5 (10.6-12.4)	12.1 (11.3-12.9)	0.4	3.4	.44	−0.2	−1.9	.97	0.6	5.4	.30
Overweight/obesity (10-17 y)	31.2 (29.8-32.6)	31.2 (29.5-33.0)	33.1 (31.6-34.7)	1.9	6.2	.18	0.0	0.1	.77	1.9	6.0	.12
Any special health care needs	19.4 (18.6-20.1)	19.0 (18.2-19.9)	19.7 (18.9-20.5)	0.3	1.7	.14	−0.3	−1.6	.93	0.6	3.4	.26
**Positive health behaviors**												
Adequate sleep (4 mo-17 y)	65.9 (64.9-66.9)	65.2 (64.0-66.4)	65.6 (64.5-66.7)	−0.2	−0.4	.91	−0.6	−1.0	.61	0.4	0.6	.72
Daily reading (0-5 y)	37.7 (36.1-39.4)	35.1 (33.1-37.1)	38.1 (36.3-39.9)	0.4	0.9	.26	−2.7	−7.0	.01	3.0	8.6	.06
Daily physical activity (6-17 y)	24.2 (23.1-25.3)	21.4 (20.3-22.6)	19.8 (18.9-20.8)	−4.4	−18.0	<.001	−2.7	−11.2	.04	−1.6	−7.6	.04

Abbreviations: ADHD, attention-deficit/hyperactivity disorder; adj, adjusted.

^a^ See eTable 2 in the Supplement for 2017 and 2018 estimates.

^b^ Adjusted models controlled for child sex, age, race and ethnicity, and household income.

### Children’s Health Care Access and Utilization

Between 2016 and 2020, there was a significant increase in the proportion of uninsured children (6.1% [95% CI, 5.5-6.7] to 7.2% [95% CI, 6.6-7.9]; 19% increase; *P* = .004) and a significant decrease in the proportion of children with adequate and continuous insurance (69.4% [95% CI, 68.4-70.3] to 67.4% [95% CI, 66.4-68.4]; 3% decrease; *P* = .004) (Table 2). Before the pandemic, there was a significant increase in children whose parents had problems paying their medical bills (15.6% [95% CI, 14.8-16.4] to 17.0% [95% CI, 15.9-18.2]; 9% increase; *P* = .04); however, in 2020, the prevalence of medical hardship dropped to the lowest rate since 2016 (13.7% [95% CI, 12.9-14.6], a 20% decrease from 2019; *P* < .001). Between 2016 and 2020, there was also a significant increase in reports of unmet needs for health care (3.0% [95% CI, 2.6-3.3] to 4.0% [95% CI, 3.5-4.6]; 36% increase; *P* = .004); this trend was driven by a 32% increase in unmet needs between 2019 and 2020 (*P* = .007). There was also a significant decrease in the proportion of children with a usual source of sick care (79.7% [95% CI, 78.7-80.6] in 2016 to 74.7% [95% CI, 73.7-75.7] in 2020, 6% decrease; *P* < .001), a trend that began prior to 2020 and worsened moderately in 2020 but was not statistically significant.

**Table 2.** Unadjusted Prevalence and Adjusted Trends for Children’s Health Care Access and Service Utilization, 2016-2020 (N = 174 551)

	Weighted prevalence, % (95% CI)^a^			5-y Trends (2016-2020)			Before COVID-19 (2016-2019)			COVID-19 era (2020 vs 2019)		
	2016 (n = 50 212)	2019 (n = 29 433)	2020 (n = 42 777)									
				Absolute difference	Relative difference	*P* value for trend (adj)^b^	Absolute difference	Relative difference	*P* value for trend (adj)^b^	Absolute difference	Relative difference	*P* value for trend (adj)^b^
**Health care access**												
Currently uninsured	6.1 (5.5-6.7)	6.8 (6.1-7.6)	7.2 (6.6-7.9)	1.2	19.0	.004	0.7	12.1	.06	0.4	6.2	.36
Adequate and continuous health insurance	69.4 (68.4-70.3)	66.0 (64.8-67.2)	67.4 (66.4-68.4)	−2.0	−2.9	.004	−3.4	−4.9	<.001	1.4	2.1	.06
Problems paying child’s medical bills, past 12 mo	15.6 (14.8-16.4)	17.0 (15.9-18.2)	13.7 (12.9-14.6)	−1.9	−12.1	.25	1.4	9.1	.047	−3.3	−19.5	<.001
Unmet needs for health care, past 12 mo	3.0 (2.6-3.3)	3.1 (2.6-3.6)	4.0 (3.5-4.6)	1.1	36.2	.004	0.1	3.4	.58	1.0	31.8	.007
Frustrated in getting services for child, past 12 mo	17.0 (16.2-17.8)	18.0 (17.0-19.0)	17.3 (16.5-18.2)	0.3	1.9	.15	1.0	6.1	.14	−0.7	−3.9	.32
Usual source of sick care	79.7 (78.7-80.6)	75.9 (74.7-77.1)	74.7 (73.7-75.7)	−5.0	−6.3	<.001	−3.8	−4.7	<.001	−1.2	−1.6	.07
**Health service utilization (past 12 mo)**												
Preventive medical visit	78.9 (77.8-80.0)	81.0 (79.7-82.3)	74.1 (72.9-75.3)	−4.8	−6.1	<.001	2.1	2.7	.01	−6.9	−8.5	<.001
Preventive dental visit (1-17 y)	78.7 (77.8-79.6)	80.1 (79.0-81.2)	74.9 (73.9-75.8)	−3.9	−4.9	<.001	1.4	1.8	.40	−5.3	−6.6	<.001
Specialty care when needed	88.6 (86.8-90.2)	87.4 (85.0-89.5)	87.9 (85.7-89.8)	−0.7	−0.8	.19	−1.3	−1.4	.30	0.5	0.6	.81
Mental health treatment/counseling when needed (3-17 y)	82.2 (80.0-84.3)	82.7 (79.8-85.3)	79.9 (77.1-82.3)	−2.4	−2.9	.74	0.5	0.6	.81	−2.9	−3.5	.38
Developmental screening (9-35 mo)	30.4 (28.0-32.9)	37.7 (34.0-41.4)	36.1 (33.2-39.1)	5.7	18.7	<.001	7.2	23.7	.001	−1.5	−4.1	.52

Abbreviation: adj, adjusted.

^a^ See eTable 2 in the Supplement for 2017 and 2018 estimates.

^b^ Adjusted models controlled for child sex, age, race and ethnicity, and household income.

There was a slight but statistically significant increase in receipt of annual preventive medical visits in the years preceding the pandemic’s onset (78.9% [95% CI, 77.8-80.0] in 2016 to 81.0% [95% CI, 79.7-82.3] in 2019; 3% increase; *P* = .02) (Table 2 and Figure 1). However, in 2020, rates of preventive medical visits decreased significantly, to 74.1% (95% CI, 72.9-75.3), resulting in a net 6% decrease between 2016 and 2020 (*P* < .001). In addition, rates of preventive dental visits were stable between 2016 and 2019, but dropped to 74.9% (95% CI, 73.9-75.8) in 2020 from 80.1% (95% CI, 79.0-81.2) in 2019, leading to a net 5% decrease over the past 5 years (*P* < .001). There was a significant increase in developmental screenings among children aged 9 to 35 months between 2016 and 2020 (30.4% [95% CI, 28.0-32.9] to 36.1% [95% CI, 33.2-39.1]; 19% increase; *P* < .001). The upward trend in developmental screenings was driven by improvements between 2016 and 2019. There were no statistically significant changes over time with respect to frustration obtaining medical services, use of specialty care, or use of mental health care.

### Family Well-being and Stressors

**Figure 2.**
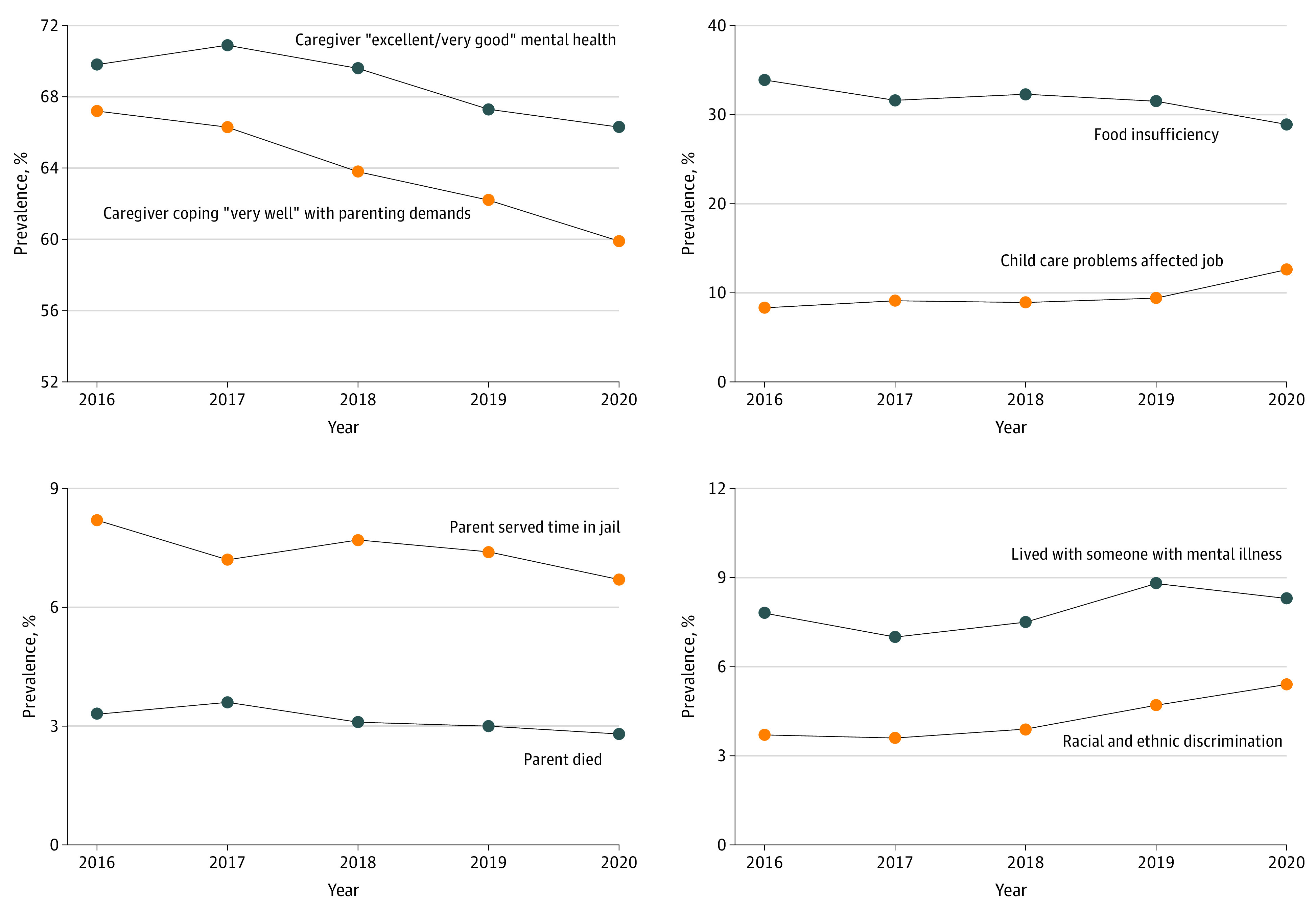
Trends in Selected Measures of Family Well-being and Stressors, 2016-2020

Between 2016 and 2020, there were significant decreases in the proportion of children with parents or caregivers in “excellent or very good” mental health (69.8% [95% CI, 68.9-70.8] to 66.3% [95% CI, 65.3-67.3]; 5% decrease; *P* < .001) and whose parents or caregivers reported coping “very well” with the demands of raising children (67.2% [95% CI, 66.3-68.1] to 59.9% [95% CI, 58.8-60.9]; 11% decrease; *P* < .001) (Table 3 and Figure 2). Both the trends for decreasing mental health and parental coping began pre-2020; there was a continued decrease in 2020 for both measures, but only the decrease in parental coping was statistically significant.

**Table 3.** Unadjusted Prevalence and Adjusted Trends for Family Well-being and Stressors, 2016-2020 (N = 174 551)

	Weighted prevalence, % (95% CI)^a^			5-y Trends (2016-2020)			Before COVID-19 (2016-2019)			COVID-19 era (2020 vs 2019)		
	2016 (n = 50 212)	2019 (n = 29 433)	2020 (n = 42 777)	Absolute difference	Relative difference	*P* value for trend (adj)^b^	Absolute difference	Relative difference	*P* value for trend (adj)^b^	Absolute difference	Relative difference	*P* value for trend (adj)^b^
**Parent or caregiver report**												
Physical health “excellent/very good”	58.8 (57.8-59.8)	58.1 (56.9-59.4)	59.0 (58.0-60.1)	0.2	0.4	.24	−0.7	−1.1	.15	0.9	1.5	.33
Mental health “excellent/very good”	69.8 (68.9-70.8)	67.3 (66.1-68.5)	66.3 (65.3-67.3)	−3.5	−5.0	<.001	−2.5	−3.6	<.001	−1.0	−1.5	.19
Coping “very well” with demands of raising children	67.2 (66.3-68.1)	62.2 (61.0-63.4)	59.9 (58.8-60.9)	−7.3	−10.9	<.001	−5.0	−7.5	<.001	−2.3	−3.8	.005
Quit, declined, changed job due to child care problems in past 12 mo (0-5 y)	8.3 (7.3-9.3)	9.4 (8.0-10.9)	12.6 (11.2-14.1)	4.3	52.1	<.001	1.1	13.5	.19	3.2	34.0	.001
Household food insufficiency in past 12 mo	33.9 (32.9-34.9)	31.5 (30.3-32.7)	28.9 (27.9-29.9)	−5.0	−14.8	<.001	−2.4	−7.2	.09	−2.6	−8.2	.003
**Child’s adverse childhood experiences**												
Parent died	3.3 (3.0-3.7)	3.0 (2.6-3.5)	2.8 (2.5-3.2)	−0.5	−15.2	.01	−0.3	−9.7	.12	−0.2	−6.1	.52
Parent served time in jail	8.2 (7.6-8.8)	7.4 (6.8-8.0)	6.7 (6.2-7.2)	−1.5	−18.3	.03	−0.8	−9.7	.42	−0.7	−9.5	.13
Witnessed interpersonal violence	5.7 (5.3-6.2)	5.6 (5.0-6.2)	5.3 (4.8-5.8)	−0.5	−7.9	.90	−0.1	−2.5	.57	−0.3	−5.5	.51
Experienced or witnessed neighborhood violence	3.9 (3.5-4.3)	4.1 (3.6-4.7)	4.1 (3.6-4.6)	0.3	6.5	.16	0.2	6.1	.20	0.0	0.4	.98
Lived with someone with mental illness	7.8 (7.3-8.3)	8.8 (8.1-9.6)	8.3 (7.7-8.9)	0.4	5.5	.002	1.0	12.4	.009	−0.5	−6.1	.29
Lived with someone with substance use problems	9.0 (8.5-9.6)	8.8 (8.0-9.6)	8.5 (7.9-9.2)	−0.5	−6.0	.97	−0.3	−3.2	.87	−0.2	−2.8	.65
Experienced racial or ethnic discrimination	3.7 (3.3-4.1)	4.7 (4.1-5.3)	5.4 (4.9-6.0)	1.7	46.7	<.001	1.0	26.1	.01	0.8	16.3	.08

Abbreviation: adj, adjusted.

^a^ See eTable 2 in the Supplement for 2017 and 2018 estimates.

^b^ Adjusted models controlled for child sex, age, race and ethnicity, and household income.

In the past 5 years, there was a significant increase in the proportion of young children whose parents quit a job, declined a job, or changed jobs because of child care problems (8.3% [95% CI, 7.3-9.3] to 12.6% [95% CI, 11.2-14.1]; 52% increase; *P* < .001); this trend was not statistically significant between 2016 and 2019, but rather was driven by a 34% increase between 2019 and 2020 (*P* = .001). There was also a significant decrease in food insufficiency between 2016 and 2020 (33.9% [95% CI, 32.9-34.9] to 28.9% [95% CI, 27.9-29.9]; 15% decrease; *P* < .001), including a notable decrease between 2019 and 2020.

There were significant decreases between 2016 and 2020 in the proportion of children who were reported to have experienced parental death during their lifetime (3.3% [95% CI, 3.0-3.7] to 2.8% [95% CI, 2.5-3.2]; 15% decrease; *P* = .01) and parental incarceration (8.2% [95% CI, 7.6-8.8] to 6.7% [95% CI, 6.2-7.2]; 18% decrease; *P* = .03) in their lifetime. Between 2016 and 2020, there were significant increases in the proportion of children who ever lived with someone with mental illness (7.8% [95% CI, 7.3-8.3] to 8.3% [95% CI, 7.7-8.9]; 6% increase; *P* = .002) and who experienced racial or ethnic discrimination (3.7% [95% CI, 3.3-4.1] to 5.4% [95% CI, 4.9-6.0]; 47% increase; *P* < .001); trends for both measures were evident before the onset of the pandemic. There were no statistically significant changes over the 5-year period in the prevalence of parent or caregiver physical health and certain lifetime adverse childhood experiences among children (interpersonal violence, neighborhood violence, living with someone with substance use problems).

### Sensitivity Analyses

After pooling the data from 2016 to 2019 and comparing with 2020, results were unchanged for 18 of 22 measures examined (data available on request). The following measures showed no significant changes in the original analysis but did show significant changes in the pooled analysis: asthma (decrease), current uninsurance (increase), parental death (decrease), and parental incarceration (decrease).

## Discussion

Information about recent trends in US children’s health and health care is needed to inform future research, clinical care, policy decision making, and programmatic investments. This analysis provides an opportunity to evaluate the nation’s progress (or lack thereof) in improving the health and well-being of US children and their families, including the first opportunity to use the NSCH to investigate potential effects of the COVID-19 pandemic.

With respect to prepandemic trends, there was a significant increase in diagnosed mental health conditions, specifically a 27% increase in anxiety and a 24% increase in depression, between 2016 and 2019. These findings are consistent with reports from other data sources.^23,24,25,26^ The direction of these trends continued into 2020, representing 5.6 million children with diagnosed anxiety and 2.4 million children diagnosed with depression; although the year-over-year increases were not statistically significant in this analysis, other data sources based on electronic health records and surveillance programs have indicated that the pandemic exacerbated said trends.^27,28^ In addition, we found a 21% year-over-year uptick in diagnoses of behavior or conduct problems from 2019 to 2020, representing about 5 million children in 2020, consistent with other parent reports that children have been “acting out” more since the start of the pandemic.^29^ Despite the increasing mental health needs of children, this study found no significant improvement in receipt of mental health treatment or counseling over the past 5 years; as of 2020, only 80% of children who needed mental health care received any services. Furthermore, we found a steady decline over the past 5 years in parent or caregiver well-being (as reflected by self-reports about mental and emotional health and coping with parenting demands) and an increase in the proportion of children who ever lived with someone with mental health problems. Results suggest that difficulties coping with parenting demands were also exacerbated by the pandemic. These findings mirror other reports of heightened stress among US adults and especially parents, both before and during the COVID-19 pandemic.^30,31,32,33^ Between 2019 and 2020, there was also a 34% increase in the proportion of young children whose parents quit, declined, or changed jobs because of child care problems; child care problems were reported for 13% of young children in 2020, representing more than 2.8 million children. Other federal data have shown that 18% of households with children reported child care disruptions more than 1 year after the onset of the pandemic; among those households, 1 in 4 adults cut their work hours or took unpaid leave to care for children and 1 in 6 left a job or did not look for a job so they could care for children.^14^ Taken together, these findings highlight a critical need to support both children and their caregivers to improve families’ mental and emotional well-being and to provide child care options that can ensure families’ economic well-being.

Study findings also confirm previous reports that children’s health care use dropped after the pandemic’s onset.^34,35,36,37^ Specifically, in 2020, there was a 9% year-over-year decrease in preventive medical visits, a 7% decrease in preventive dental visits, and a 32% increase in unmet needs for health care. Although the prevalence of problems paying children’s medical bills had been increasing prior to the pandemic, there was a 20% drop in medical hardship from 2019 to 2020, possibly because families were delaying or were unable to access health care services. Other sources indicate that the most common reasons for missed or delayed preventive visits included limited appointment availability, health care locations being closed, and caregiver concerns about visiting health care professionals.^15^ Efforts are needed to help families make up lost ground with respect to forgone health care during the pandemic.

One positive finding pertaining to health services was the increased proportion of young children receiving developmental screenings, which increased 24% prior to the pandemic, consistent with pediatricians’ increased reports of using developmental screening tools between 2002 and 2016.^38^ However, we found that the prevalence of developmental screenings in 2019 was only 38%, indicating room for improvement. This study’s parent-reported screening prevalence in 2016 (30%) was about half that of pediatrician reports in the same year (63%),^38^ suggesting that parents may not recognize that their child received a screening. Pediatricians may also overestimate the extent to which they conduct discussions of screening results with parents.^39^

Despite the many challenges faced by US children and parents or caregivers during the pandemic, results also indicate areas of resilience. For instance, the proportion of children getting adequate sleep remained steady in 2020, and the proportion of young children who were read to every day experienced a 9% uptick (although this was not statistically significant). In addition, household food insufficiency decreased by 8% between 2019 and 2020, and none of the adverse childhood experiences we examined showed a significant worsening after the onset of the pandemic.

### Limitations

There are several study limitations to consider. First, the data do not allow causal inferences about the effects of the COVID-19 pandemic on children’s health and well-being. The 2020 NSCH was fielded several months after the pandemic began (June 2020-January 2021) and some survey items (eg, health care utilization questions) had a 12-month look-back period going as far back as June 2019. As a result, estimates produced from the 2020 NSCH may not fully capture the dynamic effect of the pandemic on children and families. Cautious interpretation of the 2020 estimates is warranted, and additional years of data are needed to determine whether 2020 was truly a turning point for certain trends and how long the indirect effects of the pandemic may last. There may also be nonresponse bias if survey respondents were systematically different from nonresponders. Nonresponse bias analyses are conducted every year for the NSCH to identify potential sources of bias and assess the degree to which survey weight adjustments reduce any identified bias. These analyses have found no strong or consistent evidence of nonresponse bias after survey weights are applied.^40^ Overall trends reported here may mask different patterns within subpopulations. Additional analyses are planned to examine the extent to which disparities between sociodemographic groups of interest have changed over the past 5 years.

More work remains to achieve the nation’s goals to improve children’s health and well-being.^9^ The findings of this study can be used to inform programmatic investments and priorities, and support stakeholders in making data-informed decisions. For instance, the Maternal and Child Health Bureau of the Health Resources and Services Administration, the federal agency that sponsors the NSCH, also administers several programs that address some of the significant health-related challenges highlighted here. The Pediatric Mental Health Care Access Program expands access to pediatric mental health care by integrating telehealth services into pediatric practices in states, territories, and tribal regions to support primary care clinicians to diagnose, treat, and refer children and youth for mental health conditions. The Maternal, Infant, and Early Childhood Home Visiting Program addresses parental stress and promotes family well-being by supporting people during pregnancy and the early childhood years; health, social service, and child development resources and skill development are offered through regular home visits to address families’ wide-ranging needs. The Bright Futures Program disseminates age-specific, evidence-based guidelines for comprehensive well-child visits, including guidelines on developmental screening and surveillance; behavioral, social, and emotional assessment; and screening for maternal postpartum depression. In response to the decline in children’s preventive care during the pandemic, the Maternal and Child Health Bureau launched the Promoting Pediatric Primary Prevention (P4) Challenge to accelerate well-child visits and immunizations in primary care settings through such innovative approaches as text-message reminders, peer-to-peer social media campaigns, mobile and pop-up clinics, and integration of primary care services into dental care.

## Conclusions

Study findings point to several areas of concern, including troubling trends that were evident before the pandemic and new challenges that arose in 2020. More analyses are needed to elucidate varying patterns within subpopulations of interest. This study adds to the growing literature pointing to an exacerbation of challenges brought on by the COVID-19 pandemic, highlighting the urgent need to ensure children’s access to timely health care services, promote healthy behaviors, and support parents to strengthen family well-being.

## Notes

Supplement. **eTable 1.** Survey Items Used in Measures for Children’s Health Conditions, Positive Health Behaviors, Health Care Access/Utilization, and Family Well-being and Stressors, 2016-2020 National Survey of Children’s Health **eTable 2.** Unadjusted Frequency and Prevalence of Child Health Conditions, Positive Health Behaviors, Health Care Access/Utilization, and Family Well-being, 2016-2020 (N=174,551)
