# Supplementary material for: Five-Year Trends in US Children’s Health and Well-being, 2016-2020
Source: JAMA Pediatr. 2022 Mar 14;176(7):e220056. doi: 10.1001/jamapediatrics.2022.0056 (PMC8922203; doi:10.1001/jamapediatrics.2022.0056)
Supplement: Supplement. — eTable 1. Survey Items Used in Measures for Children’s Health Conditions, Positive Health Behaviors, Health Care Access/Utilization, and Family Well-being and Stressors, 2016-2020 National Survey of Children’s Health eTable 2. Unadjusted Frequency and Prevalence of Child Health Conditions, Positive Health Behaviors, Health Care Access/Utilization, and Family Well-being, 2016-2020 (N=174,551) [file jamapediatr-e220056-s001.pdf]

Supplemental Online Content

Lebrun-Harris LA, Ghandour RM, Kogan MD, Warren MD. Five-year trends in US children's health and well-being, 2016-2020. *JAMA Pediatr*. Published online March 14, 2022. doi:10.1001/jamapediatrics.2022.0056

eTable 1. Survey Items Used in Measures for Children's Health Conditions, Positive Health Behaviors, Health Care Access/Utilization, and Family Well-being and Stressors, 2016-2020 National Survey of Children's Health

eTable 2. Unadjusted Frequency and Prevalence of Child Health Conditions, Positive Health Behaviors, Health Care Access/Utilization, and Family Well-being, 2016-2020 (N=174,551)

This supplemental material has been provided by the authors to give readers additional information about their work.

© 2022 American Medical Association. All rights reserved.

eTable 1: Survey Items Used in Measures for Children's Health Conditions, Positive Health Behaviors, Health Care Access/Utilization, and Family Well-being and Stressors, 2016-2020 National Survey of Children's Health

MEASURES	SURVEY ITEMS	RESPONSE OPTIONS	AGE GROUP (AND SCORING METHOD FOR MULTI-ITEM MEASURES)
CURRENT HEALTH CONDITIONS	Has a doctor or other health care provider EVER told you that this child has…		
**Asthma**	Asthma?	Yes<br>No	0-17 years
	If yes, does this child currently have the condition?	Yes<br>No	
**Headaches/Migraines**	Frequent or severe headaches, including migraine?	Yes<br>No	3-17 years
	If yes, does this child currently have the condition?	Yes<br>No	
**Anxiety Problems**	Anxiety Problems?	Yes<br>No	3-17 years
	If yes, does this child currently have the condition?	Yes<br>No	
**Depression**	Depression?	Yes<br>No	3-17 years
	If yes, does this child currently have the condition?	Yes<br>No	
**Behavioral/Conduct Problems**	Behavioral or Conduct Problems?	Yes<br>No	3-17 years
	If yes, does this child currently have the condition?	Yes<br>No	
**Autism**	Autism or Autism Spectrum Disorder (ASD)? *Include diagnoses of Asperger's Disorder or Pervasive Developmental Disorder (PDD).*	Yes<br>No	3-17 years

© 2022 American Medical Association. All rights reserved.

MEASURES	SURVEY ITEMS	RESPONSE OPTIONS	AGE GROUP (AND SCORING METHOD FOR MULTI-ITEM MEASURES)
	If yes, does this child currently have the condition?	Yes<br>No	
**ADHD**	Attention Deficit Disorder or Attention Deficit/Hyperactivity Disorder, that is, ADD or ADHD?	Yes<br>No	3-17 years
	If yes, does this child currently have the condition?	Yes<br>No	
**Decayed Teeth or Cavities**	During the past 12 months, has this child had FREQUENT or CHRONIC difficulty with any of the following?<br>Decayed teeth or cavities	Yes<br>No	1-17 years
**Overweight/Obesity**	What is this child's current height?	___ feet AND ___ inches<br>OR<br>___ meters AND<br>___ centimeters	10-17 years<br><br>Child height and weight are used to calculate body-mass index and identify children who are overweight or obese (at or above the 85th percentile). This indicator is limited to children ages 10-17 years because height and weight data reported in the NSCH are only considered reliable for those older ages.
	How much does this child currently weigh?	___ pounds OR ___ kilograms	
**Any Special Health Care Needs**	**Prescription Medication:**<br>Does this child CURRENTLY need or use medicine prescribed by a doctor, other than vitamins?	Yes<br>No	0-17 years<br><br>Special health care needs are defined if parents answer "Yes" to all the items under one or more of the five qualifying criteria (prescription medication; elevated use of services; functional limitations; specialized therapy; ongoing emotional, development, or behavioral conditions).

© 2022 American Medical Association. All rights reserved.

MEASURES	SURVEY ITEMS	RESPONSE OPTIONS	AGE GROUP (AND SCORING METHOD FOR MULTI-ITEM MEASURES)
	If yes, is this child's need for prescription medicine because of ANY medical, behavioral, or other health condition?	Yes<br>No	
	If yes, is this a condition that has lasted or is expected to last 12 months or longer?	Yes<br>No	
	**Elevated Use of Services:**<br>Does this child need or use more medical care, mental health, or educational services than is usual for most children of the same age?	Yes<br>No	
	If yes, is this child's need for medical care, mental health, or educational services because of ANY medical, behavioral, or other health condition?	Yes<br>No	
	If yes, is this a condition that has lasted or is expected to last 12 months or longer?	Yes<br>No	
	**Functional Limitations:**<br>Is this child limited or prevented in any way in his or her ability to do the things most children of the same age can do?	Yes<br>No	
	If yes, is this child's limitation in abilities because of ANY medical, behavioral, or other health condition?	Yes<br>No	
	If yes, is this a condition that has lasted or is expected to last 12 months or longer?	Yes<br>No	

© 2022 American Medical Association. All rights reserved.

MEASURES	SURVEY ITEMS	RESPONSE OPTIONS	AGE GROUP<br>(AND SCORING METHOD FOR MULTI-ITEM MEASURES)
	**Specialized Therapy:**<br>Does this child need or get special therapy, such as physical, occupational, or speech therapy?	Yes<br>No	
	If yes, is this because of ANY medical, behavioral, or other health condition?	Yes<br>No	
	If yes, is this a condition that has lasted or is expected to last 12 months or longer?	Yes<br>No	
	**Ongoing Emotional, Development, or Behavioral Conditions:**<br>Does this child have any kind of emotional, developmental, or behavioral problem for which he or she needs treatment or counseling?	Yes<br>No	
	If yes, has his or her emotional, developmental, or behavioral problem lasted or is it expected to last 12 months or longer?	Yes<br>No	
POSITIVE HEALTH BEHAVIORS			
**Adequate Sleep**	(0-5 years) During the past week, how many hours of sleep did this child get during an average day (count both nighttime sleep and naps)?	Less than 7 hours<br>7 hours<br>8 hours<br>9 hours<br>10 hours<br>11 hours<br>12 or more hours	4 months-17 years<br><br>Adequate sleep is defined as children who meet the following age-specific sleep recommendations:<br>4 months-11 months: 12-16 hours<br>1-2 years: 11-14 hours<br>3-5 years: 10-13 hours

© 2022 American Medical Association. All rights reserved.

MEASURES	SURVEY ITEMS	RESPONSE OPTIONS	AGE GROUP (AND SCORING METHOD FOR MULTI-ITEM MEASURES)
	(6-17 years) During the past week, how many hours of sleep did this child get on most weeknights?	Less than 6 hours<br>6 hours<br>7 hours<br>8 hours<br>9 hours<br>10 hours<br>11 or more hours	6-12 years: 9-12 hours<br>13-17 years: 8-10 hours
**Daily Reading**	During the past week, how many days did you or other family members read to this child?	0 days<br>1-3 days<br>4-6 days<br>Every day	0-5 years
**Daily Physical Activity**	During the past week, on how many days did this child exercise, play a sport, or participate in physical activity for at least 60 minutes?	0 days<br>1-3 days<br>4-6 days<br>Every day	6-17 years
**HEALTH CARE ACCESS**			
**Currently Uninsured**	Is this child CURRENTLY covered by ANY kind of health insurance or health coverage plan?	Yes<br>No	0-17 years<br><br>Current lack of health insurance is derived from the questions on any current insurance coverage and type of coverage. Children with only Indian Health Service but no other insurance coverage are coded as uninsured. In addition, other health insurance write-in responses are back-coded to categorize insurance; coverage through a health care sharing ministry only is considered uninsured.
	Is this child CURRENTLY covered by any of the following types of health insurance or health coverage plans? *Mark (X) Yes or No for EACH item.*		
	Insurance through a current or former employer or union	Yes<br>No	
	Insurance purchased directly from an insurance company	Yes<br>No	

© 2022 American Medical Association. All rights reserved.

MEASURES	SURVEY ITEMS	RESPONSE OPTIONS	AGE GROUP (AND SCORING METHOD FOR MULTI-ITEM MEASURES)
	Medicaid, Medical Assistance, or any kind of government assistance plan for those with low incomes or a disability	Yes<br>No	
	TRICARE or other military health care	Yes<br>No	
	Indian Health Service	Yes<br>No	
	Other, specify: _________________	Yes<br>No	
**Adequate and Continuous Health Insurance**	Is this child CURRENTLY covered by ANY kind of health insurance or health coverage plan?	Yes<br>No	0-17 years<br><br>Adequate and continuous insurance is defined as meeting the following 5 conditions: (a) has current health insurance, (b) had insurance for the past 12 months with no gap in coverage, (c) insurance benefits always or usually meet child's needs, (d) insurance always or usually allows child to see needed health care providers, and (e) health care costs for the child are always or usually reasonable (or there are $0 costs).
	During the past 12 months, was this child EVER covered by ANY kind of health insurance or health coverage plan?	Yes, this child was covered all 12 months<br>Yes, but this child had a gap in coverage<br>No	
	How often does this child’s health insurance offer benefits or cover services that meet this child’s needs?	Always<br>Usually<br>Sometimes<br>Never	
	How often does this child’s health insurance allow him or her to see the health care providers he or she needs?	Always<br>Usually<br>Sometimes<br>Never	

© 2022 American Medical Association. All rights reserved.

MEASURES	SURVEY ITEMS	RESPONSE OPTIONS	AGE GROUP (AND SCORING METHOD FOR MULTI-ITEM MEASURES)
	Including co-pays and amounts from Health Savings Accounts (HSA) and Flexible Spending Accounts (FSA), how much money did you pay for this child's medical, health, dental, and vision care during the past 12 months? *Do not include health insurance premiums or costs that were or will be reimbursed by insurance or another source.*	$0 (No medical or health-related expenses)<br>$1-$249<br>$500-$999<br>$1,000-$5,000<br>More than $5,000	
	How often are these costs reasonable?	Always<br>Usually<br>Sometimes<br>Never	
**Problems Paying Child's Medical Bills**	During the past 12 months, did your family have problems paying for any of this child's medical or health care bills?	Yes<br>No	0-17 years
**Unmet Needs for Health Care**	During the past 12 months, was there any time when this child needed health care but it was not received? *By health care, we mean medical care as well as other kinds of care like dental care, vision care, and mental health services.*	Yes<br>No	0-17 years
**Frustrated in Getting Services for Child**	During the past 12 months, how often were you frustrated in your efforts to get services for this child?	Never<br>Sometimes<br>Usually<br>Always	0-17 years<br><br>Frustration is defined as parents reporting that they were "Always", "Usually", or "Sometimes" frustrated in the past 12 months.

© 2022 American Medical Association. All rights reserved.

MEASURES	SURVEY ITEMS	RESPONSE OPTIONS	AGE GROUP (AND SCORING METHOD FOR MULTI-ITEM MEASURES)
**Usual Source of Sick Care**	Is there a place that this child USUALLY goes when he or she is sick or you or another caregiver needs advice about his or her health?	Yes<br>No	0-17 years
	If yes, where does this child USUALLY go first?	Doctor's office<br>Hospital emergency room<br>Hospital outpatient department<br>Clinic or health center<br>Retail store clinic or "Minute Clinic"<br>School (nurse's office, athletic trainer's office)<br>Some other place	0-17 years<br><br>Usual source of sick care is defined has having a place the child usually goes to when sick or in need of advice, which is NOT the hospital emergency room.
**HEALTH SERVICE UTILIZATION**			
**Preventive Medical Visit**	During the past 12 months, did this child see a doctor, nurse, or other health care professional for sick-child care, well-child check-ups, physical exams, hospitalizations or any other kind of medical care?	Yes<br>No	0-17 years<br><br>Preventive medical visits are defined as 1 or more visits for a preventive check-up in the past 12 months.

© 2022 American Medical Association. All rights reserved.

MEASURES	SURVEY ITEMS	RESPONSE OPTIONS	AGE GROUP (AND SCORING METHOD FOR MULTI-ITEM MEASURES)
	If yes, during the past 12 months, how many times did this child visit a doctor, nurse, or other health care professional to receive a PREVENTIVE check-up? *A preventive check-up is when this child was not sick or injured,*<br>*such as an annual or sports physical, or well-child visit.*	0 visits<br>1 visit<br>2 or more visit	
**Preventive Dental Visit**	During the past 12 months, did this child see a dentist or other oral health care provider for any kind of dental or oral health care?	Yes, saw a dentist<br>Yes, saw other oral health care provider<br>No	1-17 years<br><br>Preventive dental visits are defined as 1 or more visits for preventive dental care in the past 12 months.
	If yes, during the past 12 months, did this child see a dentist or other oral health care provider for PREVENTIVE dental care, such as check-ups, dental cleanings, dental sealants, or fluoride treatments?	No preventive visits in the past months<br>Yes, 1 visit<br>Yes, 2 or more visits	
**Specialty Care When Needed**	During the past 12 months, did this child see a specialist other than a mental health professional? *Specialists are doctors like surgeons, heart doctors, allergy doctors, skin doctors, and others who specialize in one area of health care.*	Yes<br>No, but this child needed to see a specialist<br>No, this child did not need to see a specialist	0-17 years

© 2022 American Medical Association. All rights reserved.

MEASURES	SURVEY ITEMS	RESPONSE OPTIONS	AGE GROUP (AND SCORING METHOD FOR MULTI-ITEM MEASURES)
**Mental Health Treatment/Counseling When Needed**	During the past 12 months, has this child received any treatment or counseling from a mental health professional? *Mental health professionals include psychiatrists, psychologists, psychiatric nurses, and clinical social workers.*	Yes<br>No, but this child needed to see a mental health professional<br>No, this child did not need to see a mental health professional	3-17 years
**Developmental Screening**	During the past 12 months, did a doctor or other health care provider have you or another caregiver fill out a questionnaire about observations or concerns you may have about this child's development, communication, or social behaviors? *Sometimes a child's doctor or other health care provider will ask a parent to do this at home or during a child's visit.*	Yes<br>No	9-35 months<br><br>Developmental screening is defined as parents who indicate that they filled out a questionnaire about their child's development, communication, or social behavior AND that this questionnaire included 2 additional age-specific components capturing language development and social behavior.
	If yes, and this child is 9-23 Months:<br>Did the questionnaire ask about your concerns or observations about:<br>*Mark (X) ALL that apply.*	How this child talks or makes speech sounds?<br>How this child interacts with you and others?	
	If yes, and this child is 2-5 Years:<br>Did the questionnaire ask about your concerns or observations about:<br>*Mark (X) ALL that apply.*	Words and phrases this child uses and understands?<br>How this child behaves and gets along with you and others?	

© 2022 American Medical Association. All rights reserved.

MEASURES	SURVEY ITEMS	RESPONSE OPTIONS	AGE GROUP (AND SCORING METHOD FOR MULTI-ITEM MEASURES)
FAMILY WELL-BEING AND STRESSORS			
**Caregiver(s) Physical Health "Excellent" or "Very Good"**	In general, how is your physical health?	Excellent<br>Very good<br>Good<br>Fair<br>Poor	0-17 years<br><br>Caregiver physical health combines the reported physical health status of the primary caregiver responding to the survey as well as the health status of a second primary caregiver in the household (if there are two caregivers).
	In general, how is this caregiver's physical health?	Excellent<br>Very good<br>Good<br>Fair<br>Poor	
**Caregiver(s) Mental Health "Excellent" or "Very Good"**	In general, how is your mental or emotional health?	Excellent<br>Very good<br>Good<br>Fair<br>Poor	0-17 years<br><br>Caregiver mental health combines the reported mental health status of the primary caregiver responding to the survey as well as the mental health status of a second primary caregiver in the household (if there are two caregivers).
	In general, how is this caregiver's mental or emotional health?	Excellent<br>Very good<br>Good<br>Fair<br>Poor	
**Coping "Very Well" with Demands of Raising Children**	How well do you think you are handling the day-to-day demands of raising children?	Very well<br>Somewhat well<br>Not very well<br>Not well at all	0-17 years

© 2022 American Medical Association. All rights reserved.

MEASURES	SURVEY ITEMS	RESPONSE OPTIONS	AGE GROUP (AND SCORING METHOD FOR MULTI-ITEM MEASURES)
**Quit, Declined, or Changed Job Due to Child Care Problems**	During the past 12 months, did you or anyone in the family have to quit a job, not take a job, or greatly change your job because of problems with child care for this child?	Yes<br>No	0-5 years
**Household Food Insufficiency**	Which of these statements best describes your household's ability to afford the food you need during the past 12 months?	We could always afford to eat good nutritious meals.<br>We could always afford enough to eat but not always the kinds of food we should eat.<br>Sometimes we could not afford enough to eat.<br>Often we could not afford enough to eat.	0-17 years<br><br>Food insufficiency is defined as households that often or sometimes could not afford enough to eat or that had enough to eat but not always the kinds of food they wanted.
**Parent Died**	To the best of your knowledge, has this child ever experienced any of the following?<br><br>Parent or guardian died	Yes<br>No	0-17 years
**Parent Served Time in Jail**	Parent or guardian served time in jail	Yes<br>No	0-17 years
**Child Witnessed Interpersonal Violence**	Saw or heard parents or adults slap, hit, kick, punch one another in the home	Yes<br>No	0-17 years
**Child was Victim of or Witnessed Neighborhood Violence**	Was a victim of violence or witnessed violence in their neighborhood	Yes<br>No	0-17 years

© 2022 American Medical Association. All rights reserved.

MEASURES	SURVEY ITEMS	RESPONSE OPTIONS	AGE GROUP (AND SCORING METHOD FOR MULTI-ITEM MEASURES)
**Child Lived with Someone with Mental Illness**	Lived with anyone who was mentally ill, suicidal, or severely depressed	Yes<br>No	0-17 years
**Child Lived with Someone with Substance Use Problems**	Lived with anyone who had a problem with alcohol or drugs	Yes<br>No	0-17 years
**Child Experienced Racial/Ethnic Discrimination**	Treated or judged unfairly because of their race or ethnic group	Yes<br>No	0-17 years

© 2022 American Medical Association. All rights reserved.

eTable 2. Unadjusted frequency and prevalence of child health conditions, positive health behaviors, health care access/utilization, and family well-being, 2016-2020 (N=174,551)

	**2016 (N=50,212)**			**2017 (N=21,599)**		
	Est. Pop. Freq.	Wgt. %	95% CI	Est. Pop. Freq.	Wgt. %	95% CI
**CURRENT HEALTH CONDITIONS**						
Asthma	6,101,983	8.4	7.9,9.0	5,423,570	7.5	6.8,8.3
Headaches/Migraines (3-17 years)	2,163,413	3.5	3.1,4.0	1,898,202	3.1	2.7,3.5
Anxiety Problems (3-17 years)	4,354,865	7.1	6.6,7.6	4,241,090	6.9	6.3,7.5
Depression (3-17 years)	1,934,235	3.1	2.9,3.5	1,770,151	2.9	2.5,3.3
Behavioral/Conduct Problems (3-17 years)	4,508,698	7.4	6.9,7.9	4,179,189	6.8	6.1,7.6
Autism (3-17 years)	1,528,687	2.5	2.2,2.8	1,921,908	3.1	2.5,3.9
ADHD (3-17 years)	5,395,548	8.9	8.4,9.4	5,281,291	8.7	7.9,9.5
Decayed teeth/cavities (1-17 years)	8,005,636	11.7	11.0,12.4	8,100,922	11.7	10.7,12.9
Overweight/Obesity (10-17 years)	9,370,447	31.2	29.8,32.6	9,611,147	30.7	28.8,32.8
Any Special Health Care Needs	14,196,961	19.4	18.6,20.1	13,327,498	18.2	17.1,19.2
**POSITIVE HEALTH BEHAVIORS**						
Adequate Sleep (4 months-17 years)	46,401,858	65.9	64.9,66.9	45,807,346	64.1	62.7,65.6
Daily Reading (0-5 years)	8,784,775	37.7	36.1,39.4	8,983,872	38.2	35.8,40.7
Daily Physical Activity (6-17 years)	11,695,378	24.2	23.1,25.3	10,762,931	22.0	20.6,23.5
**HEALTH CARE ACCESS**						
Currently Uninsured	4,347,135	6.1	5.5,6.7	4,540,903	6.3	5.5,7.3
Adequate and Continuous Health Insurance	50,588,164	69.4	68.4,70.3	49,288,751	67.5	66.1,68.8
Problems Paying Child's Medical Bills, past 12 months	7,321,839	15.6	14.8,16.4	7,652,903	15.9	14.6,17.2
Unmet Needs for Health Care, past 12 months	2,156,914	3.0	2.6,3.3	2,246,818	3.1	2.5,3.8
Frustrated in Getting Services for Child, past 12 months	12,337,789	17.0	16.2,17.8	12,433,646	17.1	15.9,18.3
Usual Source of Sick Care	57,488,958	79.7	78.7,80.6	56,389,449	77.9	76.5,79.1

© 2022 American Medical Association. All rights reserved.

	2016 (N=50,212)			2017 (N=21,599)		
	Est. Pop. Freq.	Wgt. %	95% CI	Est. Pop. Freq.	Wgt. %	95% CI
**HEALTH SERVICE UTILIZATION (PAST 12 MONTHS)**						
Preventive Medical Visit[a]	38,382,826	78.9	77.8,80.0	38,974,133	78.3	76.7,79.9
Preventive Dental Visit (1-17 years)	54,064,831	78.7	77.8,79.6	55,474,283	80.2	78.9,81.5
Specialty Care When Needed	10,407,755	88.6	86.8,90.2	10,154,567	89.1	86.6,91.2
Mental Health Treatment/Counseling When Needed (3-17 years)	6,306,886	82.2	80.0,84.3	5,774,971	77.8	73.4,81.6
Developmental Screening (9-35 months)	2,636,434	30.4	28.0,32.9	2,722,753	31.7	28.1,35.6
**FAMILY WELL-BEING AND STRESSORS**						
Caregiver(s) Physical Health "Excellent" or "Very Good"	41,329,928	58.8	57.8,59.8	41,970,056	59.5	58.0,60.9
Caregiver(s) Mental Health "Excellent" or "Very Good"	48,863,406	69.8	68.9,70.8	49,754,514	70.9	69.5,72.2
Coping "Very Well" with Demands of Raising Children	48,625,770	67.2	66.3,68.1	48,147,200	66.3	65.0,67.7
Quit, Declined, or Changed Job Due to Child Care Problems, past 12 months (0-5 years)	1,925,864	8.3	7.3,9.3	2,123,438	9.1	7.6,10.7
Household Food Insufficiency, past 12 months	24,164,047	33.9	32.9,34.9	22,604,862	31.6	30.2,33.0
**Adverse Childhood Experiences**						
Parent Died	2,351,057	3.3	3.0,3.7	2,539,534	3.6	3.0,4.2
Parent Served Time in Jail	5,749,103	8.2	7.6,8.8	5,074,286	7.2	6.5,7.9
Child Witnessed Interpersonal Violence	4,020,228	5.7	5.3,6.2	3,498,207	5.0	4.4,5.5
Child was Victim of or Witnessed Neighborhood Violence	2,710,505	3.9	3.5,4.3	2,650,202	3.8	3.3,4.3
Child Lived with Someone with Mental Illness	5,487,519	7.8	7.3,8.3	4,956,572	7.0	6.4,7.7
Child Lived with Someone with Substance Use Problems	6,358,004	9.0	8.5,9.6	5,597,594	7.9	7.2,8.7
Child Experienced Racial/Ethnic Discrimination	2,604,679	3.7	3.3,4.1	2,569,636	3.6	3.2,4.1

© 2022 American Medical Association. All rights reserved.

eTable 2. (cont'd)

	2018 (N=30,530)			2019 (N=29,433)			2020 (N=42,777)		
	Est. Pop. Freq.	Wgt %	95% CI	Est. Pop. Freq.	Wgt %	95% CI	Est. Pop. Freq.	Wgt %	95% CI
**CURRENT HEALTH CONDITIONS**									
Asthma	5,564,467	7.7	7.1,8.3	5,637,439	7.8	7.2,8.5	5,179,486	7.2	6.7,7.7
Headaches/Migraines (3-17 years)	2,051,610	3.3	2.9,3.8	1,998,501	3.3	2.8,3.8	1,819,772	3.0	2.6,3.4
Anxiety Problems (3-17 years)	4,965,760	8.1	7.5,8.7	5,510,878	9.0	8.3,9.7	5,590,422	9.2	8.6,9.8
Depression (3-17 years)	2,271,305	3.7	3.3,4.1	2,394,958	3.9	3.4,4.4	2,444,255	4.0	3.6,4.5
Behavioral/Conduct Problems (3-17 years)	4,249,190	6.9	6.4,7.5	4,123,891	6.7	6.1,7.4	4,962,991	8.1	7.5,8.8
Autism (3-17 years)	1,703,927	2.8	2.4,3.2	1,910,025	3.1	2.7,3.6	1,670,138	2.7	2.4,3.1
ADHD (3-17 years)	5,323,384	8.8	8.1,9.4	5,276,693	8.6	8.0,9.3	5,644,182	9.3	8.7,9.9
Decayed teeth/cavities (1-17 years)	8,191,455	11.8	11.1,12.6	7,945,759	11.5	10.6,12.4	8,330,948	12.1	11.3,12.9
Overweight/Obesity (10-17 years)	9,788,634	30.8	29.2,32.5	10,045,557	31.2	29.5,33.0	10,584,129	33.1	31.6,34.7
Any Special Health Care Needs	13,819,038	18.8	18.0,19.7	13,923,683	19.0	18.2,19.9	14,328,072	19.7	18.9,20.5
**POSITIVE HEALTH BEHAVIORS**									
Adequate Sleep (4 months-17 years)	46,744,567	65.4	64.2,66.6	46,248,877	65.2	64.0,66.4	45,986,699	65.6	64.5,66.7
Daily Reading (0-5 years)	8,273,094	35.5	33.6,37.5	8,076,977	35.1	33.1,37.1	8,675,693	38.1	36.3,39.9
Daily Physical Activity (6-17 years)	11,426,870	23.2	22.0,24.5	10,457,390	21.4	20.3,22.6	9,618,828	19.8	18.9,20.8
**HEALTH CARE ACCESS**									
Currently Uninsured	4,798,802	6.7	6.0,7.4	4,876,511	6.8	6.1,7.6	5,140,024	7.2	6.6,7.9
Adequate and Continuous Health Insurance	49,380,492	67.5	66.4,68.6	48,036,886	66.0	64.8,67.2	48,708,702	67.4	66.4,68.4
Problems Paying Child's Medical Bills, past 12 months	7,011,973	14.9	14.0,15.8	8,038,959	17.0	15.9,18.2	6,360,308	13.7	12.9,14.6
Unmet Needs for Health Care, past 12 months	2,341,146	3.2	2.8,3.7	2,228,351	3.1	2.6,3.6	2,920,874	4.0	3.5,4.6
Frustrated in Getting Services for Child, past 12 months	12,093,997	16.6	15.7,17.5	13,092,554	18.0	17.0,19.0	12,508,743	17.3	16.5,18.2
Usual Source of Sick Care	54,717,013	75.4	74.3,76.6	54,770,245	75.9	74.7,77.1	53,693,757	74.7	73.7,75.7

© 2022 American Medical Association. All rights reserved.

	2018 (N=30,530)			2019 (N=29,433)			2020 (N=42,777)		
	Est. Pop. Freq.	Wgt %	95% CI	Est. Pop. Freq.	Wgt %	95% CI	Est. Pop. Freq.	Wgt %	95% CI
**HEALTH SERVICE UTILIZATION (PAST 12 MONTHS)**									
Preventive Medical Visit[a]	…	…	…	39,644,069	81.0	79.7,82.3	38,210,648	74.1	72.9,75.3
Preventive Dental Visit (1-17 years)	54,808,165	79.1	78.1,80.1	55,430,851	80.1	79.0,81.2	51,445,731	74.9	73.9,75.8
Specialty Care When Needed	9,943,771	89.4	87.0,91.4	10,177,908	87.4	85.0,89.5	9,112,328	87.9	85.7,89.8
Mental Health Treatment/Counseling When Needed (3-17 years)	6,124,958	81.7	79.0,84.1	6,863,643	82.7	79.8,85.3	6,728,029	79.9	77.1,82.3
Developmental Screening (9-35 months)	3,062,100	35.2	32.0,38.5	3,261,524	37.7	34.0,41.4	3,016,365	36.1	33.2,39.1
**FAMILY WELL-BEING AND STRESSORS**									
Caregiver(s) Physical Health "Excellent" or "Very Good"	41,488,702	59.2	57.9,60.4	40,419,147	58.1	56.9,59.4	40,979,278	59.0	58.0,60.1
Caregiver(s) Mental Health "Excellent" or "Very Good"	48,536,345	69.6	68.5,70.7	46,768,621	67.3	66.1,68.5	45,936,328	66.3	65.3,67.3
Coping "Very Well" with Demands of Raising Children	46,191,179	63.8	62.6,64.9	44,837,993	62.2	61.0,63.4	42,498,513	59.9	58.8,60.9
Quit, Declined, or Changed Job Due to Child Care Problems, past 12 months (0-5 years)	2,055,271	8.9	7.8,10.2	2,144,454	9.4	8.0,10.9	2,852,672	12.6	11.2,14.1
Household Food Insufficiency, past 12 months	23,090,569	32.3	31.1,33.4	22,358,496	31.5	30.3,32.7	20,330,537	28.9	27.9,29.9
**Adverse Childhood Experiences**									
Parent Died	2,186,035	3.1	2.7,3.5	2,103,028	3.0	2.6,3.5	1,956,290	2.8	2.5,3.2
Parent Served Time in Jail	5,411,287	7.7	7.0,8.4	5,140,776	7.4	6.8,8.0	4,611,727	6.7	6.2,7.2
Child Witnessed Interpersonal Violence	3,996,711	5.7	5.1,6.3	3,880,900	5.6	5.0,6.2	3,641,049	5.3	4.8,5.8
Child was Victim of or Witnessed Neighborhood Violence	2,951,268	4.2	3.7,4.8	2,841,616	4.1	3.6,4.7	2,833,687	4.1	3.6,4.6
Child Lived with Someone with Mental Illness	5,282,499	7.5	7.0,8.1	6,114,168	8.8	8.1,9.6	5,704,579	8.3	7.7,8.9
Child Lived with Someone with Substance Use Problems	5,756,674	8.2	7.6,8.8	6,080,149	8.8	8.0,9.6	5,874,418	8.5	7.9,9.2
Child Experienced Racial/Ethnic Discrimination	2,768,660	3.9	3.5,4.4	3,244,772	4.7	4.1,5.3	3,744,841	5.4	4.9,6.0

Abbreviations: Est Pop Freq=estimated population frequency. Wgt %=weighted prevalence. 95% CI=95% confidence intervals.

[a] Estimate for preventive medical visits not calculated for 2018 due to a change in question wording for that year. Question wording reverted to the previous version beginning in 2019.

eTable 2. Unadjusted rates of child health conditions, health care access/utilization, and family well-being, 2016-2020 (N=174,551)

© 2022 American Medical Association. All rights reserved.

	2016 (N=50,212)			2017 (N=21,599)		
	Est. Pop. Freq.	Wgt. %	95% CI	Est. Pop. Freq.	Wgt. %	95% CI
**CURRENT HEALTH CONDITIONS**						
Asthma	6,101,983	8.4	7.9,9.0	5,423,570	7.5	6.8,8.3
Headaches/Migraines (3-17 years)	2,163,413	3.5	3.1,4.0	1,898,202	3.1	2.7,3.5
Anxiety Problems (3-17 years)	4,354,865	7.1	6.6,7.6	4,241,090	6.9	6.3,7.5
Depression (3-17 years)	1,934,235	3.1	2.9,3.5	1,770,151	2.9	2.5,3.3
Behavioral/Conduct Problems (3-17 years)	4,508,698	7.4	6.9,7.9	4,179,189	6.8	6.1,7.6
Autism (3-17 years)	1,528,687	2.5	2.2,2.8	1,921,908	3.1	2.5,3.9
ADHD (3-17 years)	5,395,548	8.9	8.4,9.4	5,281,291	8.7	7.9,9.5
Decayed teeth/cavities (1-17 years)	8,005,636	11.7	11.0,12.4	8,100,922	11.7	10.7,12.9
Overweight/Obesity (10-17 years)	9,370,447	31.2	29.8,32.6	9,611,147	30.7	28.8,32.8
Any Special Health Care Needs	14,196,961	19.4	18.6,20.1	13,327,498	18.2	17.1,19.2
**POSITIVE HEALTH BEHAVIORS**						
Adequate Sleep (4 months-17 years)	46,401,858	65.9	64.9,66.9	45,807,346	64.1	62.7,65.6
Daily Reading (0-5 years)	8,784,775	37.7	36.1,39.4	8,983,872	38.2	35.8,40.7
Daily Physical Activity (6-17 years)	11,695,378	24.2	23.1,25.3	10,762,931	22.0	20.6,23.5
**HEALTH CARE ACCESS**						
Currently Uninsured	4,347,135	6.1	5.5,6.7	4,540,903	6.3	5.5,7.3
Adequate and Continuous Health Insurance	50,588,164	69.4	68.4,70.3	49,288,751	67.5	66.1,68.8
Problems Paying Child's Medical Bills, past 12 months	7,321,839	15.6	14.8,16.4	7,652,903	15.9	14.6,17.2
Unmet Needs for Health Care, past 12 months	2,156,914	3.0	2.6,3.3	2,246,818	3.1	2.5,3.8
Frustrated in Getting Services for Child, past 12 months	12,337,789	17.0	16.2,17.8	12,433,646	17.1	15.9,18.3
Usual Source of Sick Care	57,488,958	79.7	78.7,80.6	56,389,449	77.9	76.5,79.1
**HEALTH SERVICE UTILIZATION (PAST 12 MONTHS)**						

© 2022 American Medical Association. All rights reserved.

	2016 (N=50,212)			2017 (N=21,599)		
	Est. Pop. Freq.	Wgt. %	95% CI	Est. Pop. Freq.	Wgt. %	95% CI
Preventive Medical Visit[a]	38,382,826	78.9	77.8,80.0	38,974,133	78.3	76.7,79.9
Preventive Dental Visit (1-17 years)	54,064,831	78.7	77.8,79.6	55,474,283	80.2	78.9,81.5
Specialty Care When Needed	10,407,755	88.6	86.8,90.2	10,154,567	89.1	86.6,91.2
Mental Health Treatment/Counseling When Needed (3-17 years)	6,306,886	82.2	80.0,84.3	5,774,971	77.8	73.4,81.6
Developmental Screening (9-35 months)	2,636,434	30.4	28.0,32.9	2,722,753	31.7	28.1,35.6
**FAMILY WELL-BEING AND STRESSORS**						
Caregiver(s) Physical Health "Excellent" or "Very Good"	41,329,928	58.8	57.8,59.8	41,970,056	59.5	58.0,60.9
Caregiver(s) Mental Health "Excellent" or "Very Good"	48,863,406	69.8	68.9,70.8	49,754,514	70.9	69.5,72.2
Coping "Very Well" with Demands of Raising Children	48,625,770	67.2	66.3,68.1	48,147,200	66.3	65.0,67.7
Quit, Declined, or Changed Job Due to Child Care Problems, past 12 months (0-5 years)	1,925,864	8.3	7.3,9.3	2,123,438	9.1	7.6,10.7
Household Food Insufficiency, past 12 months	24,164,047	33.9	32.9,34.9	22,604,862	31.6	30.2,33.0
**Adverse Childhood Experiences**						
Parent Died	2,351,057	3.3	3.0,3.7	2,539,534	3.6	3.0,4.2
Parent Served Time in Jail	5,749,103	8.2	7.6,8.8	5,074,286	7.2	6.5,7.9
Child Witnessed Interpersonal Violence	4,020,228	5.7	5.3,6.2	3,498,207	5.0	4.4,5.5
Child was Victim of or Witnessed Neighborhood Violence	2,710,505	3.9	3.5,4.3	2,650,202	3.8	3.3,4.3
Child Lived with Someone with Mental Illness	5,487,519	7.8	7.3,8.3	4,956,572	7.0	6.4,7.7
Child Lived with Someone with Substance Use Problems	6,358,004	9.0	8.5,9.6	5,597,594	7.9	7.2,8.7
Child Experienced Racial/Ethnic Discrimination	2,604,679	3.7	3.3,4.1	2,569,636	3.6	3.2,4.1

© 2022 American Medical Association. All rights reserved.

eTable 2. (cont'd)

	2018 (N=30,530)			2019 (N=29,433)			2020 (N=42,777)		
	Est. Pop. Freq.	Wgt %	95% CI	Est. Pop. Freq.	Wgt %	95% CI	Est. Pop. Freq.	Wgt %	95% CI
**CURRENT HEALTH CONDITIONS**									
Asthma	5,564,467	7.7	7.1,8.3	5,637,439	7.8	7.2,8.5	5,179,486	7.2	6.7,7.7
Headaches/Migraines (3-17 years)	2,051,610	3.3	2.9,3.8	1,998,501	3.3	2.8,3.8	1,819,772	3.0	2.6,3.4
Anxiety Problems (3-17 years)	4,965,760	8.1	7.5,8.7	5,510,878	9.0	8.3,9.7	5,590,422	9.2	8.6,9.8
Depression (3-17 years)	2,271,305	3.7	3.3,4.1	2,394,958	3.9	3.4,4.4	2,444,255	4.0	3.6,4.5
Behavioral/Conduct Problems (3-17 years)	4,249,190	6.9	6.4,7.5	4,123,891	6.7	6.1,7.4	4,962,991	8.1	7.5,8.8
Autism (3-17 years)	1,703,927	2.8	2.4,3.2	1,910,025	3.1	2.7,3.6	1,670,138	2.7	2.4,3.1
ADHD (3-17 years)	5,323,384	8.8	8.1,9.4	5,276,693	8.6	8.0,9.3	5,644,182	9.3	8.7,9.9
Decayed teeth/cavities (1-17 years)	8,191,455	11.8	11.1,12.6	7,945,759	11.5	10.6,12.4	8,330,948	12.1	11.3,12.9
Overweight/Obesity (10-17 years)	9,788,634	30.8	29.2,32.5	10,045,557	31.2	29.5,33.0	10,584,129	33.1	31.6,34.7
Any Special Health Care Needs	13,819,038	18.8	18.0,19.7	13,923,683	19.0	18.2,19.9	14,328,072	19.7	18.9,20.5
**POSITIVE HEALTH BEHAVIORS**									
Adequate Sleep (4 months-17 years)	46,744,567	65.4	64.2,66.6	46,248,877	65.2	64.0,66.4	45,986,699	65.6	64.5,66.7
Daily Reading (0-5 years)	8,273,094	35.5	33.6,37.5	8,076,977	35.1	33.1,37.1	8,675,693	38.1	36.3,39.9
Daily Physical Activity (6-17 years)	11,426,870	23.2	22.0,24.5	10,457,390	21.4	20.3,22.6	9,618,828	19.8	18.9,20.8
**HEALTH CARE ACCESS**									
Currently Uninsured	4,798,802	6.7	6.0,7.4	4,876,511	6.8	6.1,7.6	5,140,024	7.2	6.6,7.9
Adequate and Continuous Health Insurance	49,380,492	67.5	66.4,68.6	48,036,886	66.0	64.8,67.2	48,708,702	67.4	66.4,68.4
Problems Paying Child's Medical Bills, past 12 months	7,011,973	14.9	14.0,15.8	8,038,959	17.0	15.9,18.2	6,360,308	13.7	12.9,14.6
Unmet Needs for Health Care, past 12 months	2,341,146	3.2	2.8,3.7	2,228,351	3.1	2.6,3.6	2,920,874	4.0	3.5,4.6
Frustrated in Getting Services for Child, past 12 months	12,093,997	16.6	15.7,17.5	13,092,554	18.0	17.0,19.0	12,508,743	17.3	16.5,18.2
Usual Source of Sick Care	54,717,013	75.4	74.3,76.6	54,770,245	75.9	74.7,77.1	53,693,757	74.7	73.7,75.7

© 2022 American Medical Association. All rights reserved.

	2018 (N=30,530)			2019 (N=29,433)			2020 (N=42,777)		
	Est. Pop. Freq.	Wgt %	95% CI	Est. Pop. Freq.	Wgt %	95% CI	Est. Pop. Freq.	Wgt %	95% CI
**HEALTH SERVICE UTILIZATION (PAST 12 MONTHS)**									
Preventive Medical Visit[a]	…	…	…	39,644,069	81.0	79.7,82.3	38,210,648	74.1	72.9,75.3
Preventive Dental Visit (1-17 years)	54,808,165	79.1	78.1,80.1	55,430,851	80.1	79.0,81.2	51,445,731	74.9	73.9,75.8
Specialty Care When Needed	9,943,771	89.4	87.0,91.4	10,177,908	87.4	85.0,89.5	9,112,328	87.9	85.7,89.8
Mental Health Treatment/Counseling When Needed (3-17 years)	6,124,958	81.7	79.0,84.1	6,863,643	82.7	79.8,85.3	6,728,029	79.9	77.1,82.3
Developmental Screening (9-35 months)	3,062,100	35.2	32.0,38.5	3,261,524	37.7	34.0,41.4	3,016,365	36.1	33.2,39.1
**FAMILY WELL-BEING AND STRESSORS**									
Caregiver(s) Physical Health "Excellent" or "Very Good"	41,488,702	59.2	57.9,60.4	40,419,147	58.1	56.9,59.4	40,979,278	59.0	58.0,60.1
Caregiver(s) Mental Health "Excellent" or "Very Good"	48,536,345	69.6	68.5,70.7	46,768,621	67.3	66.1,68.5	45,936,328	66.3	65.3,67.3
Coping "Very Well" with Demands of Raising Children	46,191,179	63.8	62.6,64.9	44,837,993	62.2	61.0,63.4	42,498,513	59.9	58.8,60.9
Quit, Declined, or Changed Job Due to Child Care Problems, past 12 months (0-5 years)	2,055,271	8.9	7.8,10.2	2,144,454	9.4	8.0,10.9	2,852,672	12.6	11.2,14.1
Household Food Insufficiency, past 12 months	23,090,569	32.3	31.1,33.4	22,358,496	31.5	30.3,32.7	20,330,537	28.9	27.9,29.9
**Adverse Childhood Experiences**									
Parent Died	2,186,035	3.1	2.7,3.5	2,103,028	3.0	2.6,3.5	1,956,290	2.8	2.5,3.2
Parent Served Time in Jail	5,411,287	7.7	7.0,8.4	5,140,776	7.4	6.8,8.0	4,611,727	6.7	6.2,7.2
Child Witnessed Interpersonal Violence	3,996,711	5.7	5.1,6.3	3,880,900	5.6	5.0,6.2	3,641,049	5.3	4.8,5.8
Child was Victim of or Witnessed Neighborhood Violence	2,951,268	4.2	3.7,4.8	2,841,616	4.1	3.6,4.7	2,833,687	4.1	3.6,4.6
Child Lived with Someone with Mental Illness	5,282,499	7.5	7.0,8.1	6,114,168	8.8	8.1,9.6	5,704,579	8.3	7.7,8.9
Child Lived with Someone with Substance Use Problems	5,756,674	8.2	7.6,8.8	6,080,149	8.8	8.0,9.6	5,874,418	8.5	7.9,9.2
Child Experienced Racial/Ethnic Discrimination	2,768,660	3.9	3.5,4.4	3,244,772	4.7	4.1,5.3	3,744,841	5.4	4.9,6.0

Abbreviations: Est Pop Freq=estimated population frequency. Wgt %=weighted prevalence. 95% CI=95% confidence intervals.

[a] Estimate for preventive medical visits not calculated for 2018 due to a change in question wording for that year. Question wording reverted to the previous version beginning in 2019.

© 2022 American Medical Association. All rights reserved.
